# Social Structure of Lions (*Panthera leo*) Is Affected by Management in Pendjari Biosphere Reserve, Benin

**DOI:** 10.1371/journal.pone.0084674

**Published:** 2014-01-08

**Authors:** Etotépé A. Sogbohossou, Hans Bauer, Andrew Loveridge, Paul J. Funston, Geert R. De Snoo, Brice Sinsin, Hans H. De Iongh

**Affiliations:** 1 Laboratory of Applied Ecology, University of Abomey-Calavi, Cotonou, Benin; 2 Wildlife Conservation Research Unit, Zoology, University of Oxford, Tubney, United Kingdom; 3 Panthera, Kongola, Caprivi Region, Namibia; 4 Institute for Environmental Sciences, University of Leiden, Leiden, The Netherlands; University of Tasmania, Australia

## Abstract

Lion populations have undergone a severe decline in West Africa. As baseline for conservation management, we assessed the group structure of lions in the Pendjari Biosphere Reserve in Benin. This reserve, composed of one National Park and two Hunting Zones, is part of the WAP transboundary complex of protected areas. Overall mean group size was 2.6±1.7 individuals (n = 296), it was significantly higher in the National Park (2.7±1.7, n = 168) than in the Hunting Zones (2.2±1.5, n = 128). Overall adult sex ratio was even, but significantly biased towards females (0.67) in the National Park and towards males (1.67) in the Hunting Zones. Our results suggest that the Pendjari lion population is affected by perturbations, such as trophy hunting.

## Introduction

Lions *Panthera leo* are the most gregarious of all felids, forming ‘fission-fusion’ social units known as prides that typically comprise four to six (range 1–21) related females, their dependent offspring and a temporary, unrelated coalition of typically two (range 1–9) adult males [1]–[4]. Prides rarely move collectively, encounters in the field are usually with subunits that we refer to as groups. Several factors influence lion grouping patterns, such as cub defence, group territoriality, defence of kills against scavengers, synchronised female breeding patterns and communal raising of offspring [1], [4]–[6]. External factors such as anthropogenic pressures also affect the lion grouping pattern and social behaviour [7], [8].

Lion social behaviour varies across its range [9], [10]. In West and Central Africa, lion populations have severely declined [11], [12], with densities below 5 lions/100 km^2^ [13]. Lions in this region tend to form small groups [14]. Lions are Regionally Endangered [15] and genetically distinct [16] making ecological research in West Africa relevant and urgent [17].

Here we present data on the group structure of the lion population in Pendjari Biosphere Reserve, Benin. This West African reserve is part of the W-Arly-Pendjari (WAP) complex of protected areas across three countries: the ‘W’ National Park (NP) in Benin, Burkina Faso and Niger, Arly Reserve in Burkina Faso and Pendjari Biosphere Reserve in Benin. Lions occur throughout the complex but at lower densities in W NP (P. Henschel unpublished data) where habitat is still intact but where livestock numbers are very high [18] (P. Bouché unpublished data). Like most parts of West Africa, WAP is affected by habitat degradation and fragmentation, hunting and illegal grazing. Grazing inside the WAP is probably the biggest perturbation, but this threat has not been systematically monitored. Lion hunting quotas in Benin were halved after the first lion population census in 2002 [19]. Currently lion hunting quota is six lions every two years in Pendjari and four lions every two years in W Benin. Lion hunting is not allowed in Niger, but in Burkina Faso the quota exceeds 20 and effective offtake has been about 12 lions per year [20].

## Methods

### Study area

**Figure 1:**
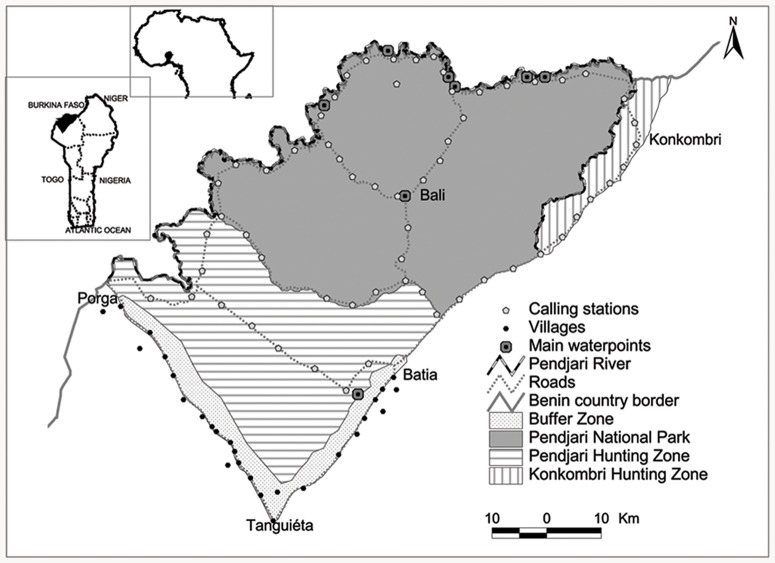
Location of Pendjari Biosphere Reserve in in north-west Benin comprising one National Park, two Hunting Zones and a buffer zone.

The study was conducted in Pendjari Biosphere Reserve (Fig. 1). The reserve consists of Pendjari NP (2,660 km^2^), Pendjari Hunting Zone (HZ) (1,600 km^2^) and Konkombri HZ (250 km^2^). The climate in Pendjari is characterized by one dry season (November-May) and one rainy season (May-October). Rainfall varies from 800 mm in the North to 1,000 mm in the South and mean temperature ranges from 18.6°C to 36.8°C. Most rivers and waterholes dry up between February and May with water available only in parts of the Pendjari River and a few important natural waterholes. In the rainy season, many areas of the reserve are flooded and inaccessible. The vegetation is a mosaic of savannah, floodplains and gallery forest [21]. The mammalian fauna is characteristic of the West African savannah including lion, leopard *Panthera pardus*, cheetah *Acinonys jubatus*, spotted hyaena *Crocuta crocuta*, and wild dog *Lycaon pictus* [21].

### Assessment of social structure

To assess social structure, we systematically searched all the existing roads by car and motorbike for at least 15 days each month during the dry seasons of 2008–2009 and 2009–2010. The Professional Hunters (PH) in charge of the HZs made observations during the same period with a similar sampling effort. Motorised transport is hardly possible off-road and during the wet season. In addition to our own and the PH's observations, we collated all sightings of tourist guides for the same years.

For our own and PH's observations, we recorded GPS coordinates, group composition, vegetation type and whether the observation was <250 m from surface water. Lions were grouped in three age classes based on the criteria of Schaller [1]: cubs (less than two years), sub-adults (two to four years) and adults (more than four years). When possible, the sex was determined. From observations by others we only used group size data, since they could have easily confused maneless males with females and subadults with adults. Individual identification is a good method to study social structure [22] but lions were too skittish to use it in Pendjari reserve.

### Data analysis

We used Kruskal Wallis (H) tests to find differences in social structure in the NP compared to the HZs.

### Ethics statement

The research did not involve invasive methods; permission was given by the authority in charge of the area (National Centre for Management of Wildlife Reserves, CENAGREF).

## Results

Our data set comprises 296 encounters with lion groups, 168 from the NP and 128 from the HZs, with a total of 763 lion observations. From this data set, 218 were our own observations and 57 from the PH; the remaining 21 observations were made by rangers or guides.

### Group sizes

The average lion group size in the entire reserve, all ages considered, was 2.6±1.7 (n = 296). The mean group size was significantly higher in the NP (2.7±1.7 lions, range 1–8, n = 168) than the HZs (2.2±1.5 lions, range 1–5, H = 6.5, df = 1; P<0.01, n = 128). The mean number of adults in mixed groups was 1.0±0.2, in male coalitions it was 1.1±0.2 (range 1–4). There was an average of 1.2±0.5 adult lionesses in groups.

**Figure 2:**
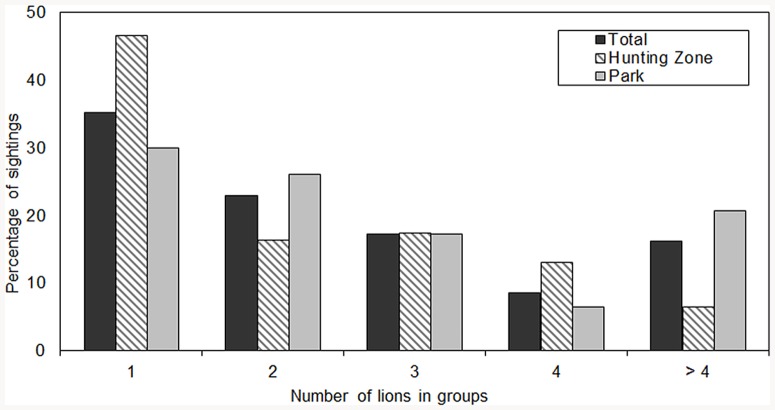
Frequency of different lion group sizes sightings in Pendjari Biosphere Reserve (n = 296 observations from 2008 to 2010).

Fig. 2 shows the frequencies of different group sizes observed in the NP and HZs. The proportion of single lion observations was significantly higher in the HZs (46.7%) compared to the NP (29.9%) (χ^2^ = 7.89, df = 1, P<0.005, n = 296). Most observations (75.3%) of groups of more than four lions were made in the NP. In the entire reserve, 64.4% of solitary individuals were adult males while 24% were adult females (rest unidentified). Most (67.6%) observations in the NP were made close to waterpoints.

### Age and sex composition

Males ∶females ratio was 1 for the entire reserve (Table 1), but we observed significantly more males than females in the HZs (ratio of 1.54, H = 11.6; df = 1; P<0.001, n = 127) while we found the opposite in the NP (ratio of 0.69, H = 20.1; df = 1; P<0.001, n = 199).

**Table 1:** Age and sex composition of lions in Pendjari Biosphere Reserve based on 763 lion observations in 296 lion group encounters in 2008–2010.

	Reserve	Hunting Zones	Park
Sex ratio adults (male ∶ female)	1∶1	1∶0.6	1∶1.5
	(158∶168)	(77∶50)	(81∶118)
Age composition (%)			
- Cubs	19.9 (n = 110)	25.0 (n = 50)	16.9 (n = 60)
- Subadult	7.9 (n = 44)	8.0 (n = 16)	7.9 (n = 28)
- Adult	72.2 (n = 401)	67.0 (n = 134)	75.2 (n = 267)

About 20% of the lion population were cubs (Table 1); there was no significant difference between the proportion of cubs (H = 0.58; df = 1; P = 0.45, n = 110) and sub-adults (H = 1.79; df = 1; P = 0.18, n = 44) in the NP and the HZs. The number of cubs in groups varied from one to six with a mean of 3.8.

## Discussion

In literature, most lion populations have a sex ratio skewed towards females and a higher proportion of immature lions (typically around 40–50%) [7], [23]–[25]; at this stage we cannot satisfactorily explain why the values observed in our study area appear to be different. It could be an artefact related to their more secretive behaviour; we tried to avoid this bias by having a large dataset but we are aware that the population is small and there is some degree of pseudo-replication. However, the female: cub ratio showed that the population had the potential to reproduce effectively.

Social structure in the HZs was markedly different from that in the NP: the HZ had significantly smaller groups, significantly more observations of single lions and a significantly different sex ratio skewed towards males. Considering that the HZ and the NP are very similar in all biophysical aspects, we infer that management in the HZ leads to reduced sociality, but we refrain from speculating about the ecological pathways that lead to this reduced sociality.

Low lion density in Pendjari and most countries of West and Central Africa is accompanied by small group size; Bauer *et al.* [9] suggested three hypotheses: low mean prey body size, low prey density and dependence of lions on livestock. Alternatively, lions may be less inclined to form larger groups in areas where low lion density reduces intergroup conflict [26]. Larger male coalitions have greater success in pride take-overs and longer tenure times [27], but the rarity of large male coalitions in Pendjari may further reduce the need for larger groups. However, the reverse argument can also be made: with low density and small group sizes, competition between males for prides may be relatively low.

In Pendjari, lions are not dependent on livestock [28]. In support of the group territoriality hypothesis [4], lions in Kgalagadi form larger stable prides when they have young cubs and then fragment into subgroups as the cubs get older [26]. In Zimbabwe, Loveridge *et al.* [8] found that prides living on the edges of the PA and thus exposed to more anthropogenic pressure than prides in the core areas are characterized by a low female group size and low cub survival. The latter is consistent with increased frequency of male takeovers and subsequent infanticide associated with male removal, leading to low cub rates at moderate offtake levels. In contrast, we found substantially (but not significantly) higher cub rates in HZs; this could be due to excessive male removal leading to female prides being unattended by males for extended periods and thus reduced infanticide [8]. Alternatively, but also indicative of excessive removal, it could be that persecution of sub-adults and adults make for proportionally higher cub rates. While speculations on cub rates are not conclusive, our other results infer that anthropogenic disturbance and mortality through trophy hunting and persecution may be important drivers of low lion density and small group size in Pendjari, and in other protected areas in West and Central Africa [8], [11], [29].

We have no data on lion poaching or poisoning, but we suspect that it occurs. The hunting quota of 6 per two years is never achieved, only one or two males are hunted per year and it would be unwise to increase the quota based on the difficulty to find suitable lion trophies (Sogbohossou, pers. obs.). Packer *et al.* [30] suggested quotas of 0.5 lion/1000 km^2^ and recommended shooting only males over 6 years old; Pendjari quota are three times higher and the existing regulation defining only ‘old males’ as eligible trophies is not enforced. Lions from the NP probably fill gaps created by hunting and poaching in Benin and Burkina Faso, comparable to the ‘vacuum effect’ described by Loveridge *et al*. [8], [31].

### Suggestions for conservation

Against the declines across West and Central Africa, the apparently stable Pendjari or even WAP lion population represents a unique stronghold. In view of the high quota in Burkina Faso, investigations on a transboundary scale are needed to better appraise the impact of trophy hunting on the lion population. Efforts should also be made to fight poaching and grazing and to improve monitoring by park staff. Reliable longitudinal data on prey density and distribution will help to understand changes in the lion population.
